# Intestinal Parasitic Infestation in Combatants and Their Families: A Hospital-Based Study in Mid-Western Regional Police Hospital, Nepal

**DOI:** 10.5539/gjhs.v6n3p9

**Published:** 2014-01-23

**Authors:** Damodar Paudel, Myo Nyein Aung, Bindhya Sharma, Thin Nyein Nyein Aung, Saiyud Moolphate

**Affiliations:** 1 Mid-West Regional Police Hospital, Nepalgunj, Nepal; 2 Department of Public Health, Juntendo University Graduate School of Medicine, Tokyo, Japan; 3 Boromarajonani College of Nursing, Nakhon Lampang, (BCNLP), Thailand; 4 Department of Public Health, Faculty of Science and Technology, Chaingmai Rajabhat University, Chaing Mai, Thailand; 5 Christian-Albrechts University, Kiel, Germany; 6 University of Medicine, Mandalay, Myanmar

**Keywords:** intestinal parasitic infestation, helminth, protozoa, Ascaris, Nepal, Nepalgunj

## Abstract

**Objective:**

To find out the scenario of intestinal parasitic infestation in combatants and their families in the setting of Mid-Western Regional Police Hospital (MWRPH), Nepal.

**Study Design:**

Cross-sectional study.

**Methods:**

All 2005 patients presented with the complaint of abdominal pain, diarrhoea, frequent defecation, blood in stool, or black stool from August 2007 to February 2011 were offered a stool examination. About 10g of fresh stool was collected in a clean, dry bottle. Two slides from each specimen were examined applying light microscope in 10 and 40 uvf at Banke, Nepalgunj hospital laboratory.

**Result:**

Among 2005 patients, 928 (46.28%) were infested with either helminths and/or protozoa. 96% were single infestation. The most common infestation was *Ascaris lumbricoides* (48.06%) and the second was hook worm (18.97%). Most common protozoal infestations were *Entamoeba histolytica* (12.92%) and *Giardia lamblia* (9.49%). Helminthic infestations peaked in cool months and protozoal infestations were rather steady throughout the year.

**Conclusion:**

Very high parasitic infestation in least developed mid- western Nepal may need urgent public health intervention.

## 1. Introduction

Parasitic diseases caused by helminthes and protozoa are common and current health problems in tropics. Soil-transmitted helminthic infections (STH) are very common in the poorest countries where sanitation is poor and human faeces contaminate the soil. Two billion people are affected by single or multiple soil transmitted helminthic diseases and 135,000 died annually (WHO, 2013). Likewise, protozoal infections are common in people living in places with poor sanitation, unsafe water and limited access to basic health care (Gunduz, Demirel, Inceboz, Tosun, & Yereli, 2005).

The global burden by disability-adjusted-life year (DALY) of soil transmitted helminthic diseases is comparable to tuberculosis and malaria (Jex et al., 2011). Creeping impact of these parasitic infestations insidiously affects women and children’s health, reduce work productivity of adults and thus, impair economic growth of poor countries (WHO, 2012). Worm infestations cause anemia and poor pregnancy outcome in women and, malnutrition, poor physical growth, and psychological cognitive underdevelopment in children (Anderson, 1982; Yap et al., 2012). Those impacts are often less visible and usually have a low priority. Nepal is a poor country with high burden of intestinal parasitic diseases in children, adult and elderly population (Gyawali, Amatya, & Nepal, 2009; Shakya, Rai, Singh, & Shrestha, 2006).

The aim of our study is to find out the seasonal trends of parasitic infections in Nepalese police combatant and their families in mid-western region of Nepal. The findings aimed to alert the respective authorities to strengthen effective control and intervention measures in study site region which has lowest human development index in the country.

## 2. Methods

This cross-sectional study was conducted in Midwestern Regional Police Hospital (MWRPH), Karkado, Banke district, Nepal. Ethical clearance was obtained by the permission of hospital. This hospital provides free health service to the servicing polices, retired polices and their families.

We performed stool examination of 2005 patients from August 2007 to February 2011. Stool examination was confined to the patients presented to MWRPH with the complaint of abdominal pain, diarrhoea, frequent defecation, blood in stool, or black stool.

Stool examination Procedure: Ten gram of fresh or routine stool was collected in a clean, dry capped plastic container. The consistency (solid, semisolid or liquid) and the color of the stool were recorded. Wet mount method was applied; small amount of stool was mounted in one drop of normal saline with a wooden stick. Mounted stool was covered with cover slip and the bubble was omitted. Each specimen was examined under a light microscope at ×10 for the helminthes ova and the cover slip was slightly pressed and removed excess liquid ×40 objectives with immersion oil seen for protozoa’s.

Data analysis applied STATA version 11. Descriptive statistics, such as percentages, mean or median, standard deviation or interquartile range, were used to summarize the data. Chi-square test or Fisher’s exact test were used to detect differences for comparing proportions of category variables. T-test was used to detect differences for continuous variables with normal distribution. P value less than 0.05 was taken as statistic significant. Age groups were categorized as in WHO soil transmitted helminthic (STH) infection report (WHO, 2012).

## 3. Results

Median age of police combatants and their family members was 25 years, ranging from the youngest being 0.98 year to the oldest 75 years old. Most of the patients were male (Table 1). Interquartile range respectively for male and female patients were 22-32 and 20-38 years, while the median age for both gender was 25 years.

**Table 1:** Characteristic of the study population

	Number	Percentage
Total (N)	2005	100
Female	389	19.40
Male	1605	80.05
Age Median (min-max))	25 (0.98-75)	
0-4	71	3.54
5-14	88	4.39
15 and above	1846	92.07
Rank		
Helper	65	3.24
Constable	1204	60.05
Junior officer	267	13.32
Senior officer	33	1.65
Retired police or, Family member*	429	21.40

* means family members of combatants

Among 2005 cases screened, 928 patients (46.28.3%) were infested with parasites in any of three forms: cyst, trophozoite or ova.

### 3.1 Infestation Rate

Overall, 46.28% of the screened patients were infested with helminths or protozoa or both (Table 2). Infestation rate was different across the age groups. School going age (5-14 years) group were most infested (54.55%) while preschool children aged 0-5 years were less infested (39.44%). Infestation rate among the adolescent and adult (age ≥15 years group) was 46.15%.

**Table 2:** Intestinal parasitic infection among combatants and their families in mid-western region of Nepal (2007-2011) by different categories

	Total N	Infestation rate n(%)	Mixed Infection n(%)	Helminthic Infection n (%)	Protozoal Infection n (%)
Total	2005	928(46.28)	77(8.30)	640(68.97)	211(22.74)
Age (years)					
0-4	71	28(39.44)	3(10.71)	16(57.14)	9(32.14)
5-14	88	48(54.55)	3(6.25)	30(62.50)	15(31.25)
15 and above	1846	852(46.15)	71(8.33)	594(69.72)	187(21.95)
Gender					
male	1605	725(45.17)	55(7.59)	497(68.55)	173(23.86)
female	389	197(50.64)	21(10.66)	139(70.56)	37(18.78)
Rank		*			
Helper	65	29 (44.62)	2(6.90)	18(62.07)	9(31.03)
Constable	1204	527(43.77)	42(7.97)	364(69.07)	121(22.96)
Junior officer	267	142(53.18)	9(6.34)	106(74.65)	27(19.01)
Senior officer	33	9(27.27)	0(0.00)	6(66.67)	3(33.33)
Retired police or, Family member	429	216(50.35)	23(10.65)	143(66.20)	50(23.15)

Note

* Infestation rate among five different categories of ranks was significantly different. Pearson chi^2^ = 17.67 P= 0.003

Infestation rate among female patients were higher than that among the male. Moreover, the infestation rate among non-gazetted junior officers was significantly higher than the other ranked combatants. Infestation rate was statistically different within the different categories of rank significantly (Pearson chi^2^ = 17.67, P-value = 0.003). However, the distribution of mixed infection, helminthic and protozoal infections were not different statistically among any categories (Table 2).

### 3.2 Spectrum of Parasitic Infestation

*Ascaris lumbricoides* was the most common infestation, the second was hook worm infestation, the third was *Entamoeba histolytica* (12.92%), the fourth was *Giardia lamblia* (9.49%) and the fifth was mixed protozoal and helminthic infection (Table 2). The number and proportion of helminthic infestation were much more than the protozoal infection. Almost half of infestations were Ascaris and almost one fifth of them were hookworm. Detail distributions of infecting parasites were shown in Table 3.

**Table 3:** Spectrum of intestinal parasitic infestation among combatants and their families in mid –western region of Nepal

Types Infestation			n	% of infestations	% of number screened
*Ascaris lumbricoides*			446	48.06	22.24
*Ancylostoma duodenale (hook worm)*			176	18.97	8.78
*Trichuris trichiura*			3	0.32	0.15
*Hymenolepis nana*			15	1.62	0.75
*Entamoeba histolytica* cyst			108	11.64	5.39
*Entamoeba histolyticaTrophozoites*			10	1.08	0.50
*Giardia lamblia* cyst			69	7.44	3.44
*Giardia lamblia* trophozoites			19	2.05	0.94
*Trichomonas intestinalis*			4	0.43	0.20
*Trichomonas homonis*			1	0.11	0.05
Mixed helminthic and protozoa infection			77	8.30	3.84
**Types of mixed infestations (n=77)**	**n=(77)**	**%**			
*Entamoeba histolytica and Giardia lamblia*	4	5.19		0.43	
*Entamoeba histolyticaand hook worm dual infection*	12	15.58		1.29	
*Entamoeba histolytica and Hymenolepis nana*	4	5.19		0.43	
*Entamoeba histolytica and Ascaris lumbricoides*	25	32.47		2.69	
*Ascaris lumbricoides and Giardia lamblia*	22	28.57		2.37	
*Ascaris lumbricoides and hookworm*	10	12.99		1.08	
**Total infestation**			928	100	46.28
**No infestation**			1077		53.72
Total	2005		2005		100

### 3.3 Annual Trend and Seasonal Variation of the Parasitosis

Parasitic infestation rate among the patients presented to MWRPH with the complaint of abdominal pain, diarrhoea, frequent defecation, blood in stool, or black stool were persistently high year-round. Helminthic infection ranged from lowest (20%) in April to highest 42.75% in November. Protozoal infection ranged from the lowest of (4.73%) in December to highest of (18.1%) in April.

**Figure 1:**
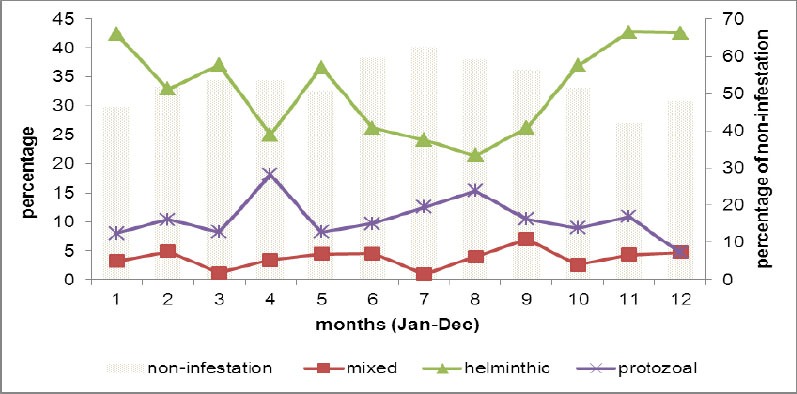
Seasonal trend of helminthic, protozoal and mixed infection throughout the year among combatants and their families, a total of 2005 persons with abdominal and bowel symptoms, in mid-western region of Nepal (2007-2011)

Monthly infestation rate of helminthic, protozoal and mixed infection were displayed in separate lines to compare the seasonal trend (Figure 1). The trend of helminthic infestation began to rise in September, continued throughout autumn until the highest in November (42.75%), December (42.6%) and January (42.4%). Afterwards, it declined in summer months until the lowest in August, with a small peak in May.

Protozoal trend is low and stable year round. Its peaks in April and August coincide with troughs of the helminthic infestation. It gradually declined to the lowest in winter months of the years: December, January and February (Figure 1). Overall, the number of mixed infections is lower than the helminthic and protozoal infection. Its trend was low in March and July, while overall trend of mixed infestations peaked in September. It was generally stable at less than 10% throughout the year.

## 4. Discussion

Overall, prevalence of parasitic infestation rate in this study was much higher (46.28%) than those reported in previous studies (Gunduz et al., 2005; Gyawali et al., 2009; Khanal, Rai, Khanal, & Ghimire, 2011). It underscored the high burden of intestinal parasitic infection especially STH in the mid-western region of Nepal and the need of multiple interventions.

The majority of the current study population was adults. Thus, the finding might reflect the burden of helminthic and protozoal infection in working age group in mid-western region of Nepal. Moreover, current study included women and children who were the family members of policemen (Table 1).

The infestation rate of overall parasitosis was higher among the women and 70% of those infestations were helminthic infection (Table 2). A previous study of enteropathogens in diarrheal stool samples in Kathmandu, Nepal reported gender indifference (Ono et al., 2001). However, helminthic infection was not included in that previous report. Therefore, helminthic infestation and its burden in mid-western region of Nepal could be the reason for the gender difference in infestation rate reported by current study finding. Given that helminthic infestation rate was higher among the female and the interquartile range of age among the female was 20-38 years, it could worsen prevalence of iron deficiency anemia among Nepalese women and result in poor pregnancy outcomes (Christian, Khatry, & West, 2004; Larocque, Casapia, Gotuzzo, & Gyorkos, 2005; Marahatta, 2007). The women living in the poverty pockets may have low knowledge of the diseases. Culturally tailored, gender sensitive interventions are necessary.

Among the different age groups, parasitic infestation was highest in school going aged children (5-14 years) (Table 2). Helminthic infestation was found in more than 60% of the cases and the remaining 30% was protozoal infestation. This finding was concurrent with previous study finding in western region of Nepal (Mukhopadhyay, Wilson, Chawla, VS, & Shivananda, 2008). Playing in contact with soil might cause contaminated with egg or larva of *A lumbricoides* and hook worm. Worm infestation in school going age results in poor school performance and absenteeism (WHO, 2012). It can cause the long term under-development of psychomotor and physical growth (Oberhelman et al., 1998). A national strategic plan of six monthly anthelminthic intervention targeting for children under 5 years of age has been started years ago in Nepal, but the impact on school children were not known yet (WHO, 2008).

Helminthic infestations were influenced by socio-economic status of the community (Lancet, 2004). In the current study, rank of the policemen reflects their living standard. Junior officers were infested most (Table 2). Sanitation facility differed among the families by rank. Unpaved barrack and the barefoot walking habit enhance the chance of STH such as ascariasis and hookworm.

Transmission of worms and parasites rely on people behavior of toileting, hand washing and nail trimming of children (Gyawali et al., 2009; Shrestha, Narayan, & Sharma, 2012). Recent WHO UNICEF update on progress of drinking water and sanitation 2012 reported that population practicing open defecation was as high as 57% in the rural area of Nepal in 2010. Until recently, 28% of households had no latrine and 27% had temporary pit latrine and 46% had private latrine with septic tank in the study site area, Nepalgunj municipality (Asian Development Bank, 2011). Such situation maintains contamination of the soil with human faeces and favours transmission dynamic of STH (Brooker, Clements, & Bundy, 2006; Lancet, 2004).

In the current study a seasonal trend of helminthic infestation, protozoa infestation and mixed infection were sorted by data collected for three years. Helminthic trend peaked in autumn (September to November) (Figure 1). Free-living infective stages of soil transmitted helminths such as *A lumbricoides* and *T trichiura* and their maximum development occur at 28° to 32°C (Beer, 1976). Temperature less than 5°C and more than 38^0^ are unfavorable for the development. Similarly hookworm ova could not resist the temperature more than 40^0^ centigrade, *A lumbricoides* eggs are more resistant to extreme temperatures (Beer, 1976). The helminthic trend in current finding saw peaks in the month of cool weather (28° to 32°C) and troughs in extremely hot summer time which usually is as hot as 38°C. Although there is seasonal fluctuation of the STH, the prolonged life span of the adult worm (1-10) years will maintain the endemicity.

Moreover, the survival and the fastest growth of larvae and ova of the parasites depend on the humidity. The higher the humidity, the better the embryogenesis of the ova of the helminthes (Brooker et al., 2006). Land surface temperature of Banke and nearby districts ranging from 5° to 46°C is favorable for the infective stage of helminth and protozoa. Consequently higher infection intensity per host and increment of the reproductive form of the parasites will maintain infection prevalence in population (Anderson, 1982; Anderson & May, 1992; Pavlovski, 1966).

In the current study population, the number of children was less than adults. Such an age distribution may result in a low prevalence of protozoal and mixed infection unlike some previous reports in Nepal (Shrestha, et al., 2012). Moreover, our finding showed a year- round persistent trend of protozoal infestation (Figure 1). The sewage system in least developed Nepalgunj has been poor for decades (Asian Development Bank, 2011). It might contribute environmental sources of infection. The cysts of E *histolytica*, the most common type of protozoa in current place (Table 3), withstand the desiccation and changes in the humidity and the temperature. However, the reason why seasonal trends of helminthic and protozoal infection seemed opposite to each other was not explainable by literature (Figure 1).

### 4.1 Limitation

This study site hospital in poor setting had limited laboratory facility of microscopic examination. Helminthic infection could be underestimated because of simple microscopic examination, and protozoa infection could be overestimated as we could not apply *E. histolytica*–specific antigen detection or polymerase chain reaction techniques to exclude E. *dispar* cysts (Haque, Huston, Hughes, Houpt, & Petri, 2003).

## 5. Conclusion

Despite the limitations, current study would point up the burden of worm infestation among combatants, women and children in their families in a least developed area of Nepal. It calls for an environmental survey in the study area which will directly claim for an investment to improve proper sanitary system. Moreover, interventions to promote people’s knowledge, personal hygienic behavior, and sanitation facilities are necessities to abolish open defecation habit, and consequent soil contamination. The concerning agencies should start all possible interventions to improve the environment as soon as possible.
